# B cell treatments for multiple sclerosis

**DOI:** 10.1007/s00415-017-8442-y

**Published:** 2017-03-04

**Authors:** D. McLauchlan, N. P. Robertson

**Affiliations:** Institute of Psychological Medicine and Clinical, Neurosciences, Cardiff University, Cardiff, CF14 4XW UK

## Introduction

The established picture of immunological dysfunction in Multiple Sclerosis (MS) posits a central role for T cells. As a consequence, the development of disease-modifying treatments (DMTs) has been targeted at sequestering, lysing or depressing production of T cells. This approach has been generally successful in reducing relapses and surrogate imaging markers of diseases activity, but has not been so effective in delaying the onset of progressive disease, and/or disability accumulation. In addition response to even high efficacy treatments is often not complete and there remain small groups of non-responders suggesting a degree of biological diversity. As a result much remains to be achieved in the field of MS therapeutics.

Conversely the role of B cells has remained relatively under-explored. However, converging lines of evidence have suggested a key role in antibody presentation, cytokine production, meningeal inflammation, axonal degeneration and grey matter demyelination. In particular, this latter feature may offer an alternative therapeutic target for the development of therapies which might expect to have greater efficacy on later and more progressive forms of disease. This month’s journal club reviews four papers addressing the role of B cell treatments in MS.

## Ocrelizumab versus interferon beta-1a in relapsing multiple sclerosis

This paper presents composite data from two clinical trials (OPERA I and OPERA II), run concurrently in two different site groups. Ocrelizumab, a CD20 inhibitor (and hence B cell selective inhibitor) was compared with the current, standard first line therapy for MS, Interferon Beta 1a.

Patients aged 18–55, with 2 relapses within the last 2 years (or one within the last 18 months), EDSS 0–5.5 and MR imaging supportive of the diagnosis were eligible for inclusion. Patients with primary progressive disease, prior B cell treatments or disease duration longer than 10 years were excluded. Participants were assigned by 1:1 randomisation to 600 mg Ocrelizumab (24 weekly) or 44 mg of interferon beta weekly. The primary outcome was the annualised relapse rate (ARR) at 96 weeks. There were a series of secondary outcomes set hierarchically for significance testing: worsening disability (EDSS increase by 1.0 or more), total number of T2 lesions, new T2 lesions, disability improvement by 1.0 on the EDSS, pooled disability progression from 24 to 96 weeks, number of hypointense regions on T1 sequences, change in the Multiple Sclerosis Functional Composite score, percentage change in brain volume, physical component score on the SF36 and finally the proportion of patients with no evidence of clinical or imaging disease progression.

1636 patients were randomized; 87.8% of patents on Ocrelizumab and 79.7% in the interferon group completed the trial. Patients on Ocrelizumab had a 46% lower ARR in OPERA I and 47% lower in OPERA II. There was a lower rate of EDSS progression at 12 weeks (9.1 vs 13.6%) and pooled disability progression at 96 weeks was lower in the Ocrelizumab group (6.9 vs 10.5%). The MRI measures also showed a lower number of total T2 lesions and new lesions in the Ocrelizumab group. The majority of new lesions occurred early in the treatment: new lesions later in treatment were less common. In addition, there were fewer T1 hypo-intense lesions in the Ocrelizumab group.

*Comments and conclusion* Ocrelizumab has demonstrated superiority over interferon beta 1a across a range of relevant clinical and imaging measures and the effects on disability progression are particularly encouraging. However, it is worth pointing out that the ARRs are comparable with a recent observational study of Rituximab, and several randomised controlled trials. The new lesions appearing earlier in treatment suggest either a ‘wash out’ effect of disease activity, or more interestingly a change in immunomodulation.

Hauser SL et al. (2017) NEJM 376:221–234.

## Ocrelizumab versus placebo in primary progressive multiple sclerosis

There are no trials to date which have shown effect on the primary outcome in progressive MS. This paper reports a randomized controlled trial of Ocrelizumab versus placebo in MS.

Inclusion criteria for the trial were age 18–55, EDSS 3.0–6.5 at screening, disease duration less than 15 years, and positive oligoclonal bands. Patients who had previously received B cell therapies, had relapsing disease at any point, or contraindications to either MRI or steroids were all excluded. EDSS and MRI images were scored by a single blinded rater. Participants were randomized on 2:1 ratio to either 600 mg or ocrelizumab every 24 weeks, continued for a total of 5 infusions, or placebo. The primary outcome measure was percentage of patients with disability progression on the EDSS at 12 weeks and sustained for 12 weeks, hierarchical secondary endpoints were the percentage of patients with disability progression confirmed at 24 weeks in a time-to-event analysis, change in performance on the timed 25-foot walk from baseline to week 120, change in the total volume of brain lesions on T2-weighted MRI from baseline to week 120, change in brain volume from week 24 to week 120 and finally change in SF36 physical component score.

732 patients were randomised, 488 on ocrelizumab (82% completed 24 weeks), 244 participants on placebo (71% completed 24 weeks). Results demonstrated a reduction in disability progression in favour of ocrelizumab at 12 weeks (ocrelizumab 32.9 vs 39.3% placebo), which was sustained at 24 weeks (ocrelizumab 29.6 vs 35.7% placebo). In addition a greater proportion of patients on placebo had deterioration in their timed walk. The imaging data also demonstrated a reduction of lesions in the ocrelizumab group, an increase in the placebo group (−3.4 vs +7.4) and a reduction in the rate of brain volume loss in the ocrelizumab group. Infections, adverse events and serious adverse events were comparable in both groups, apart from infusion reactions, which were more common in the ocrelizumab group (39.9 vs 25.2%). Importantly, the incidence of neoplasms was higher in patients treated with ocrelizumab using data from this trail and that of the Hauser paper outlined above: (0.4/1000 patients years in ocrelizumab vs 0.2 in comparator groups i.e. MS patients on alternative treatments).

*Comments and conclusions* These results are highly encouraging, and suggest that the first treatment for primary progressive disease is on the horizon. However, although the authors point out that the OLYMPUS trial was negative, it is worth noting that pre-planned subgroup analyses suggested benefits in some groups. Furthermore, the outcome measure is rather short compared to trends seen in large-scale epidemiological work, when longer periods of follow-up are required. Finally, the neoplasm rate, albeit small, is a cause for concern and would have to be discussed with patients starting ocrelizumab.

Montalban X et al. (2017) NEJM 376:209–220.

## Rituximab in multiple sclerosis: A retrospective observational study on safety and efficacy

Previous trials of Rituximab in MS have delivered mixed results with some evidence of benefit in relapsing disease, and widespread adoption within some countries. This was an observational cohort study to assess evidence of benefit from Rituximab in relapsing-remitting disease.

This retrospective cohort study used a registry drawn from three hospitals in Sweden, with participants recruited from 2001. All patients received either 500 or 1000 mg of Rituximab every 6–12 months and had MR imaging at the same time. CD19, JCV levels and immunoglobulins were all routinely recorded, as were adverse events: deaths, malignancies, autoimmune disorders, and infections.

The study included 557 patients with relapsing-remitting disease, 198 with secondary progressive disease and 67 with primary progressive disease; 20% of patients received rituximab first line and the remainder following first line treatment with either interferon or natalizumab. Mean follow-up was 23.1 months. ARR were very low: 0.044 for relapsing-remitting disease, 0.038 for secondary progressive disease and 0.015 for primary progressive disease. EDSS remained unchanged for patients with relapsing disease, and showed small, non-significant increases in progressive disease. Analysis of imaging data revealed that new contrast enhancing lesions were visible in only 4.6% and that new lesions were common in the first 6 months of treatment. Infections and infusions were the most common adverse events, three patients developed malignancies and there were four deaths.

*Comments and conclusion* The ARR in this cohort were very low, suggesting a significant effect on the inflammatory component of disease. Although by their nature retrospective cohort studies are at risk of selection bias, in this case the cohort was mainly composed of patients who had failed first line therapy, and therefore, might be considered to have a more severe disease phenotype. In addition as seen in the ocrelizumab trials new lesions were most commonly seen shortly after treatment induction.

Salzer J et al. (2016) Neurology 87:2074–2081.

## Severe B-cell-mediated CNS disease secondary to alemtuzumab therapy

This paper describes the unusual clinical course of two patients with MS who were treated with alemtuzumab; an anti CD52 therapy; considered a high efficacy treatment which causes peripheral lymphocyte ablation via complement mediated lysis. Subsequent immune reconstitution then occurs which is thought to be beneficial for MS with specific cell types repopulating after different intervals. B cells re-constitute earliest over several months with more significant delay for CD4+ and CD8+ subsets.

The first case, a 41 year old male received alemtuzumab for severe relapsing disease. Five months following first infusion he re-presented with symptoms consistent with diffuse disease relapse: apraxia, dysarthria, cognitive impairment and an asymmetrical tetraparesis. An MR brain demonstrated multiple supratentorial ring enhancing lesions. A change of treatment to Rituximab then appeared to stabilise the disease. The second case (a 25-year-old female) presented with left sided ataxia and numbness 9 months following alemtuzumab treatment. An MR brain revealed multiple ring enhancing lesions and once again she responded well to Rituximab.

*Comments and conclusion* Lack of response to alemtuzumab is unusual and in particular the very high levels of clinical and radiological disease activity post-treatment seen in these cases. It does seem likely that the aetiology of deterioration in both cases was an exacerbation of pre-existing neuro-inflammatory disease given the response to immunosuppression. The importance of this report is to lend further support to the concept of biological diversity in MS and that a subset of patients may have B cell driven disease that may be more resistant to T cell targeted therapies which may even have the effect of exacerbating ongoing inflammatory activity.

Haghikia A et al. (2017) Lancet Neurology 16:104–106.

